# Zinc and its role in vitamin D function

**DOI:** 10.1016/j.crphys.2022.04.001

**Published:** 2022-04-30

**Authors:** Ashton Amos, Mohammed S. Razzaque

**Affiliations:** Department of Pathology, Lake Erie College of Osteopathic Medicine, Erie, PA, USA

**Keywords:** Zinc, Vitamin D, Sources, Interactions, Deficiency

## Abstract

Zinc is an essential mineral with an important relationship with vitamin D. Studies have found that reduced blood zinc levels could predict vitamin D deficiency in adolescent girls, while zinc supplementation increased vitamin D levels in postmenopausal women. In vitro studies using human peritoneal macrophages have found that zinc induced the release of calcitriol (1,25-dihydroxycholecalciferol). Zinc also acts as a cofactor for vitamin D functions, as the transcriptional activity of vitamin D-dependent genes relies on zinc to exert pleiotropic functions, including mineral ion regulation. Vitamin D could also induce zinc transporters to regulate zinc homeostasis. Together, zinc and vitamin D in adequate concentrations help maintain a healthy musculoskeletal system and beyond; however, deficiency in either of these nutrients can result in various disorders affecting almost all body systems. This brief article will focus on the role of zinc in vitamin D functions.

## 1 Introduction

Vitamin D is important for the regulation of phosphate and calcium, which are needed for optimal bone health. Vitamin D has many cofactors that are required to allow vitamin D activation to exert its functions in bone health, and immunity. These cofactors, including zinc, are used as a necessary element in vitamin D functionality. Zinc is an abundant trace mineral in the body, and is a part of over 600 enzymes, numerous proteins, and over 2500 transcription factors (King et al., 2015; Kambe et al., 2015). Zinc is essential for numerous biological processes, including cellular apoptosis, immune function, brain maturation and development, taste and smell regulation, skin and mucosal integrity, and metabolic function (Kodama et al., 2020; Baarz and Rink, 2022; Brion et al., 2021; Beigi Harchegani et al., 2020). It is used as a signaling ion to regulate gene expression, protein synthesis, DNA synthesis, and cell division. Though zinc has many other important functions not related to vitamin D, the focus of this article will be on the potential interaction between zinc and vitamin D (both ligand and vitamin D receptor: 1,25-dihydroxycholecalciferol, and VDR) and its concurrent effects on the vitamin D-dependent gene activity. This article will also explain why adequate amounts of zinc is needed for vitamin D regulation and functions.

## 2 Regulation of zinc

The body contains 2–3 g of zinc, with nearly 90% found in the muscle and bone (Wastney et al., 1986). It is bound to proteins in the plasma such as albumin, metallothionein and transferrin (Kambe et al., 2021; Harris, 1983). When checking zinc status, plasma zinc concentration is measured but is not necessarily representative of total body zinc content. This is partly due to the variable amount of zinc in the intracellular compartments, including nucleic acids, which cannot be measured in plasma samples.

Daily intake of zinc is needed because our bodies do not have a true zinc storage system (Rink and Gabriel, 2000). Zinc balance is maintained mostly by the gastrointestinal tract and partly by the excretory systems (Krebs, 2000). Zinc homeostasis is regulated heavily through gastrointestinal excretion in the feces and stored temporarily in intestinal cells, where it is eventually sloughed off to be excreted. In the gastrointestinal tract, if zinc is needed, absorption occurs, and when zinc is not required, excretion in feces occurs. Human studies are very few (Krebs, 2000), but some animal studies show that intestinal cells can maintain zinc concentrations 10-fold of normal by increasing or decreasing fecal excretion. These animal studies are suggestive, but are not entirely comparative to human systems. Renal excretion is minor compared to gastrointestinal fecal excretion, and unlike gastrointestinal excretion, it typically does not change dramatically with changes in intake (King et al., 2000). Once zinc is absorbed, it can attach to metallothionein and be stored temporarily in a small zinc pool until it is needed in the intestinal cells. Zinc can also be used in metabolic functions in the cells (Kambe et al., 2015). Zinc-specific transporters are required at the cellular level for physiologic regulation of zinc; ZIP/SLC39 and CDF/ZnT/SLC30 families of transporters are involved in zinc regulation. Different types of ZIP and ZnT transporters regulate the flow of zinc intracellularly, extracellularly, and within organelles of the cells (Jeong and Eide, 2013; Bin et al., 2018; Nishito and Kambe, 2019; Huang et al., 2005; Lopez and Kelleher, 2009).

## 3 Zinc and its sources

**Fig. 1:**
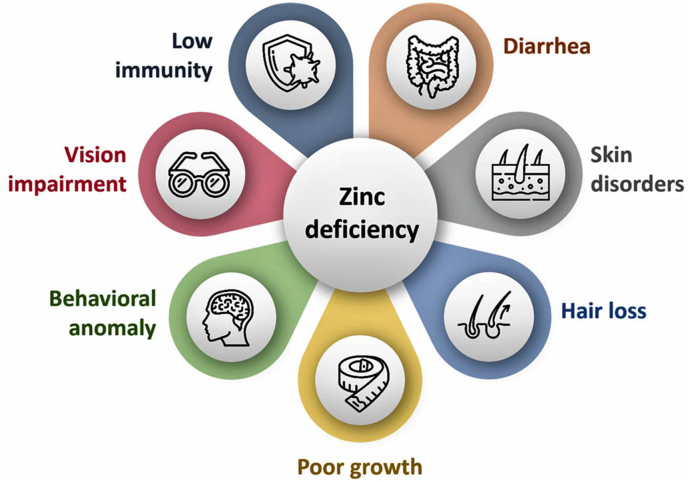
The zinc deficiency is widespread and can affect the functionality of various systems of the body.

Zinc is found naturally in foods, medications, and dietary supplements. According to a study from the Food and Agriculture Organization of the United Nations, global zinc deficiency is fairly common (Wessells and Brown, 2012). Zinc deficiency is associated with various comorbidities, including obesity, metabolic diseases, hypertension, and other cardiovascular diseases. An association between low zinc levels and the evolvement of COVID-19 is also reported (Razzaque, 2020; Razzaque, 2021). Zinc deficiency can cause growth impairment, immune dysregulation, neural system dysfunction, dermal lesions, and impaired reproductive functions (Fig. 1). Zinc deficiency is more common in certain regions, especially developing countries, but also documented in developed countries (Black, 2003; Kumssa et al., 2015). For example a study in Spain of ages ranging from 9-to 75 years old, showed that 83% of people were not meeting zinc intake requirements (Olza et al., 2017). Additionally, older adults are more prone to zinc deficiency. NHANES III (National Health and Nutrition Examination Survey) data showed that 35–45% of older adults 60 years or older were not meeting the estimated average requirement. After adjusting for supplements that most of the older population consumes, results showed inadequate zinc intake was still estimated at as high as 20–25%. In addition to elderly populations, individuals with gastrointestinal disorders, vegetarians, pregnant and lactating women, older infants who are exclusively breastfed, individuals with sickle cell disease, and those who consume excessive amounts of alcohol were also at increased risk for developing zinc deficiency (Wapnir, 2000; Temiye et al., 2011). Individuals with gastrointestinal disorders are at risk because the primary regulation of zinc is through the gastrointestinal tract, leading to either inability to absorb or increased losses of zinc related to chronic diarrheal diseases (Krebs, 2000).

Vegetarian populations are also at risk of zinc deficiency due to lower zinc bioavailability in their diets; this is partly caused by the presence of phytates. Phytates bind to zinc and inhibit absorption (Foster and Samman, 2015; Gibson et al., 2018; Segovia-Siapco et al., 2019). Food high in phytate includes cereals, legumes, oilseeds, and nuts. It has also been shown that vegetarians typically have lower zinc intakes than omnivores. Oysters, beef, crabs, and lobsters contain high sources of zinc. Pregnant and lactating women are at risk for zinc deficiency due to higher fetal requirements for zinc and zinc loss during lactation, making their recommended daily allowances higher (Donangelo and King, 2012). In a study of women of reproductive age in Ethiopia, Kenya, Nigeria, and South Africa, zinc deficiency was detected in about 34% of women; those who were pregnant ranged between 46 and 76% deficient (Harika et al., 2017). Zinc is one of the most common nutrient deficiencies in pregnant women. It is suggested to be a risk factor for adverse long-term effects on growth, immunity, and offspring survival (Gernand et al., 2016; Wang et al., 2015). The recommended daily allowance for adults is 8–12 mg/day; pregnant and lactating women need higher amounts than the general population. Older infants who are strictly breastfed are at risk, as breastmilk only contains enough zinc for infants of 4–6 months old. Infants of 7–12 months need additional dietary supplementation (National-Institute-of-Health, 2020; Uwitonze et al., 2020).

Individuals with sickle cell disease have zinc deficiency that exacerbates during sickle cell crisis. In a study of 34 sickle cell patients, lower plasma zinc concentrations and higher zinc urinary excretion were noted in patients in a steady state compared to the 50 healthy controls. During a sickle cell crisis, zinc levels dropped by a mean value of 0.54 μg/mL from a steady-state value of 0.79 μg/mL (Niell et al., 1979). Individuals who drink excessive amounts of alcohol tend to have decreased zinc absorption and increased zinc excretion abilities, compounded with less zinc intake. In addition to low consumption and absorption, functional alterations of zinc receptors contribute to zinc dyshomeostasis (McClain et al., 2017). Zinc deficiency is the most expected clinical scenario; however, zinc toxicity rarely occurs with an extremely high amount of zinc intake, causing symptoms like nausea, vomiting, and fatigue. The upper tolerable limit, the amount of a specific nutrient that one can take without the risk of developing toxicity and adverse events, is 40 mg/day for individuals from 19 years onwards for the general population (Institute-of-Medicine, 2000).

A high dose of zinc supplementation can inhibit copper uptake leading to copper deficiency from competing for the same transporters; this can lead to impaired iron absorption, anemia, leukopenia, and neutropenia (Donangelo et al., 2002; de Brito et al., 2014). Cadmium, which is increasing in the environment, can also inhibit zinc absorption. Several drugs need to be taken at separate times as zinc supplementation because, if taken together, they inhibit the absorption of the drug, zinc, or both. It has shown that zinc has inhibitory effects on different antimicrobial drugs, including quinolones and β-Lactams like Cephalexin (Ding et al., 2012; Okamura et al., 2008). Thiazide diuretics increase zinc excretion by up to 60%; therefore, these patients need to be monitored for zinc status (Wester, 1980).

## 4 Vitamin D and its sources

Vitamin D is a lipid soluble vitamin with a steroid structure. The vitamin D metabolite, also known as 25-hydroxycholecalciferol must be activated to form 1,25-dihydroxycholecalciferol to exert bioactivities. Vitamin D3 can be obtained from the sun as 7-dehydrocholesterol and converted to 25-hydroxycholecalciferol in the liver (Akimbekov et al., 2020a; Akimbekov et al., 2020b; Erem et al., 2019; Razzaque, 2011; Erem and Razzaque, 2021; Bikle et al., 2000). Whether these conversions are zinc-dependent is not yet clear. Vitamin D is necessary for bone mineralization. Due to its necessity in bone mineralization, vitamin D deficiency has been linked to an increased risk of skeletal disorders (van Driel and van Leeuwen, 2017). Vitamin D deficiency is a worldwide health concern. In certain areas, individuals do not have enough sunlight exposure to make adequate amounts of vitamin D due to their latitude and need to take vitamin D through food sources or supplementation (Erem and Razzaque, 2021; Sowah et al., 2017). Other factors that limit vitamin D absorption from the sun include darker skin pigmentation, limited sun exposure, individuals with fat malabsorption disorders, and obesity (Lo et al., 1985; Johnson et al., 2006). Both zinc and vitamin D deficiencies can be any mixture of decreased absorption, increased nutritional need, excessive excretion, or inadequate dietary intake. Vitamin D has numerous extraskeletal functions that when it becomes deficient, it can be associated with various tumors, cardiovascular disease, immunity, metabolic syndrome (obesity and diabetes), and renal diseases (Akimbekov et al., 2020a; Erem et al., 2019; Bikle et al., 2000; van Driel and van Leeuwen, 2017; Lee et al., 2019; Hossain et al., 2019).

As mentioned, vitamin D synthesis begins in the skin upon exposure to sunlight; an activated form of 1,25-dihydroxycholecalciferol is generated in the kidney, which then binds to the VDR. Ligand bound VDR then dimerizes with the retinoid X receptor (RXR) to upregulate the expression of a wide range of factors, including the calcium-binding protein (CaBP), calcium stimulated ATPase, and alkaline phosphatase, causing increased intestinal absorption of calcium and increased plasma calcium ion concentration (Craig et al., 2001). Vitamin D may not exert desired physiologic functions when zinc status is not optimal, as VDR needs zinc to regulate the functionality of vitamin D-dependent genes.

## 5 Zinc and vitamin D interactions

For 1,25-dihydroxycholecalciferol to exert biological activities, zinc is needed. VDR is similar to other steroid receptors and can specifically interact with zinc finger regions. Zinc fingers contain one or two zinc ions bound by cysteine or histidine residues and are found in many transcriptional factors, including ones contained in VDR (Matthews and Sunde, 2002). Zinc modulates the structure and binding of the DNA binding domain of the 1,25-dihydroxycholecalciferol response element DNA; therefore, without zinc, proper VDR structural conformation cannot be formed. Therefore, the activity of vitamin D-dependent genes relies on zinc, making zinc an essential cofactor for vitamin D activity (Wan et al., 2015; Craig et al., 1997; Leon and Roth, 2000). In a study on rats that were fed zinc-adequate or zinc-deficient diets, those with zinc-deficient diets showed significantly less intestinal mucosal expression of both VDR protein and CaBP protein and demonstrated that zinc deficiency has an effect on VDR expression, and functions (Yu et al., 2006). In a separate study, with increased intracellular zinc concentrations, more zinc was bound to the VDR-RXR heterodimer (Craig et al., 2001; Craig et al., 1997), indicating that zinc could influence the VDR-DNA interactions. Zinc began to dissociate at a too high concentration, again suggesting the necessity of maintaining optimal zinc balance for VDR activities. Therefore, VDR binds zinc, and the functionality of vitamin D-dependent genes is influenced by the status of zinc levels.

In a study conducted on 988 adolescent girls (12–18 years old), low blood zinc level was found to be a strong predictor of vitamin D deficiency (serum levels <20 ng) (Gonoodi et al., 2019). In a double-blind randomized trial, eight-week of zinc supplementation increased vitamin D levels in postmenopausal women (V á zquez-Lorente et al., 2021). In a similar line of study, a positive association between the serum levels of zinc and vitamin D has been documented among children and adolescents, and the odds of higher levels of vitamin D increased with higher levels of zinc (Shams et al., 2016). Hereditary vitamin D resistant rickets (HVDRR) is a rare autosomal recessive disorder with bone mineral abnormality and usually occurs in childhood. The main effect of HVDRR is defective intestinal absorption of calcium due to VDR mutations in the gastrointestinal tract (Ghazi et al., 2017).

In a study on a Brazilian family with two affected siblings, a point mutation in the first zinc finger of the DNA-binding domain of the VDR was detected. This mutation caused in a single base substitution resulting in the amino acid arginine being changed to a stop codon. The nonsense mutation resulted in a truncated protein and deleted part of the zinc fingers and ligand-binding domain. This mutation caused the siblings to have symptoms including early-onset rickets, alopecia, convulsions, hypocalcemia, secondary hyperparathyroidism, and elevated 1,25-dihydroxycholecalciferol (Mechica et al., 1997). Another study of a young French-Canadian boy showed similar symptoms due to an identical mutation (Zhu et al., 1998). The patients' parents were heterozygous for the mutant allele. In another study of a Chinese family, a different missense mutation occurred within the first zinc finger of the DNA-binding domain resulting in similar skeletal consequences (Pang et al., 2016). These three studies show the importance of the zinc finger binding domain with VDR and its effect on calcium absorption and bone mineralization. Without the functional zinc fingers, the correct transcription of vitamin D-dependent genes cannot be regulated due to resistance to 1,25-dihydroxycholecalciferol binding, leading to inhibition of any downstream effects of this ligand leading to the identified symptoms of HVDRR patients. Of biological significance, conformational changes within the DNA-binding domain of the VDR was detected upon binding with zinc (Veenstra et al., 1998).

**Fig. 2:**
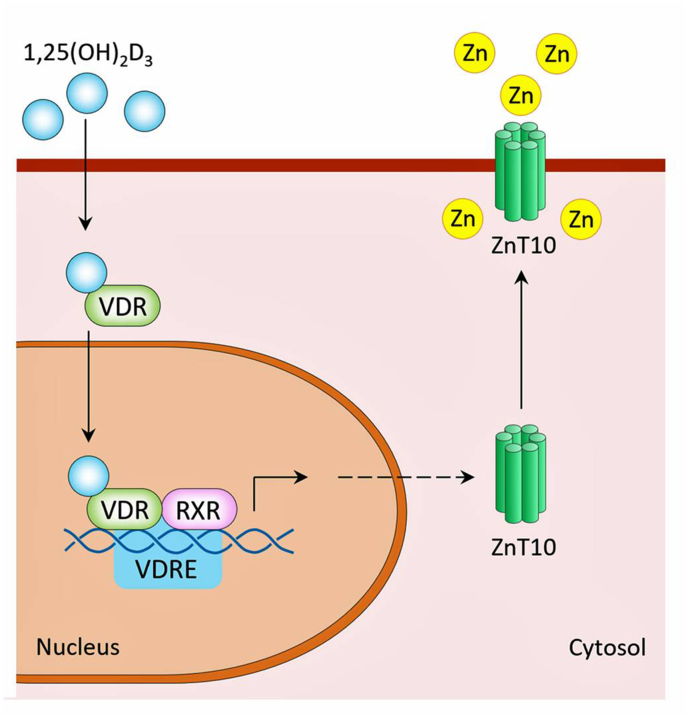
Zinc and vitamin D interactions. This figure shows how vitamin D can induce zinc transporter protein (ZnT10) to regulate zinc homeostasis (Claro da Silva et al., 2016).

Vitamin D can directly influence cellular zinc homeostasis by inducing zinc transporters. In a study of cells with vitamin D treatment, there was a 15-fold increase in the SLC30A10 gene. This gene is responsible for the translation of ZnT10 protein (Claro da Silva et al., 2016). This relationship shows that vitamin D has the ability to regulate cellular zinc homeostasis. An upregulation of ZnT10 protein allows zinc to be taken up, out of the cytosol, and increased concentrations would be available for extracellular use (Fig. 2).

## 6 Conclusion

A feed-forward loop exists between zinc and vitamin D; zinc can enhance vitamin D activities, while vitamin D can influence zinc homeostasis. Vitamin D functions are partly regulated by zinc finger-dependent transcription of vitamin D-dependent genes. When vitamin D binds to VDR, it interacts with zinc finger DNA-binding domain to regulate transcriptional activation of genes to exert cellular functions. As mentioned, zinc is an essential cofactor to have the desired functions of vitamin D. Similarly, vitamin D can also influence zinc absorption and homeostasis by regulating its transporters. Disturbance of the homeostasis of either of these nutrients can have undesirable effects leading to an abundance of disease possibilities, including but not limited to musculoskeletal disorders, cardiovascular disorders, immune dysfunction, and healing defects. The interrelationship between zinc and vitamin D is an understudied area, and further studies are needed to determine the exact molecular interactions. Determining the importance of zinc and its relationship to vitamin D is imperative in understanding the body's physiologic functions and using this information to promote preventive medicine to reduce disease burdens.

## CRediT authorship contribution statement

**Ashton Amos:** Collected information and drafted the manuscript. **Mohammed S. Razzaque:** Conceptualization, reviewed and edited the manuscript.

## Declaration of competing interest

The authors declare that they have no known competing financial interests or personal relationships that could have appeared to influence the work reported in this paper.
